# MAPK/ERK-CBP-RFPL-3 Mediates Adipose-Derived Stem Cell-Induced Tumor Growth in Breast Cancer Cells by Activating Telomerase Reverse Transcriptase Expression

**DOI:** 10.1155/2022/8540535

**Published:** 2022-06-07

**Authors:** Wenjie Li, Cheng Qian, Fei Ma, Meng Liu, Xiaojun Sun, Xu Liu, Chunxiao Liu, Zhenghua Chen, Weichang Ma, Jian Liu, Haiqian Xu, Zhenlin Yang

**Affiliations:** ^1^Department of Thyroid and Breast Surgery, Yantai Affiliated Hospital of Binzhou Medical University, Yantai, China; ^2^Department of Oncological Surgery, The Third Affiliated Hospital of Harbin Medical University, Harbin, China; ^3^Department of Breast Surgery, The Second Affiliated Hospital of Harbin Medical University, Harbin, China; ^4^Plastic and Aesthetic Surgery Center, The First Affiliated Hospital of Harbin Medical University, Harbin, China

## Abstract

Adipose-derived stem cells (ASCs) improve the self-renewal and survival of fat grafts in breast reconstruction after oncology surgery. However, ASCs have also been found to enhance breast cancer growth, and its role in tumor proliferation remains largely elusive. Here, we explored a novel mechanism that mediates hTERT reactivation during ASC-induced tumor growth in breast cancer cells. In this study, we found the proliferative ability of breast cancer cells markedly increased with ASC coculture. To explore the molecular mechanism, we treated cells with anibody/inhibitor and found that the activation of MEK-ERK pathway was triggered in breast cancer cells by SCF secreted from ASCs, leading to the nuclear recruitment of CBP. As a coactivator of hTERT, CBP subsequently coordinated with RFPL-3 upregulated hTERT transcription and telomerase activity. The inhibition of CBP and RFPL-3 abrogated the activation of hTERT transcription and the promotion of proliferation in breast cancer cells with cocultured ASCs in vitro and in vivo. Collectively, our study findings indicated that CBP coordination with RFPL-3 promotes ASC-induced breast cancer cell proliferation by anchoring to the hTERT promoter and upregulating telomerase activity, which is activated by the MAPK/ERK pathway.

## 1. Introduction

At present, adipose-derived stem cells (ASCs)-assisted lipotransfer are increasingly applied for repairing defects after breast cancer surgery because of their regenerative properties [1]. However, the results that ASCs might promote breast cancer growth have been demonstrated in our previous study [2], and the precise mechanisms that govern tumor growth are still being elucidated.

The stem cell factor (SCF)/c-kit axis plays critical roles in the tumor environment [3]. Binding of SCF to transmembrane protein c-kit causes the cascade activation of the mitogen-activated protein kinase (MAPK) and phosphatidylinositol 3-kinase (PI3K) pathways [4], in which phosphorylated extracellular-regulated protein kinase (ERK) translocates to the nucleus and stimulates transcription factor activity, including that of cAMP-response element-binding protein (CREB), which is generally involved in cell differentiation and proliferation [5, 6]. CBP, as the binding protein of CREB, can form a bridge with other transcription factors and activate transcription by acetylating histones and other regulatory proteins in tumourigenesis [7]. It has been reported that cancer-associated adipose tissue secretomes activate CBP [8]. However, at present, no data support the hypothesis that CBP is activated by the MAPK/ERK signaling pathway or coordinated with other transcription factors to regulate the ASC's tumor-promoting effect in breast cancer growth.

Telomerase, a reverse transcriptase that endows cells with unlimited chromosome replication, is thought to be crucial for the process of carcinogenesis [9]. Human telomerase reverse transcriptase (hTERT) is a critical subunit of telomerase, and its ectopic expression is involved in tumor proliferation, metastasis, and sensitivity to chemoradiotherapy [10, 11]. Accumulating evidence demonstrates that numerous transcription factors are involved in regulating the activity of the hTERT promoter [12]; however, the breast cancer-specific activation mechanisms of hTERT during tumourigenesis remain unclear. Members of the ret finger protein-like (RFPL) protein family, similar to the ret finger protein, are involved in regulating the cell cycle and embryonic development [13]. The RFPL-3 gene is located proximal to telomeres, and the protein has special structural characteristics, such as a tripartite structure and the absence of a DNA-binding domain, which enable it to mediate protein-protein interactions but not bind to target gene promoters [14]. Previous research has reported that RFPL-3 could activate the hTERT promoter and promote the growth of human lung cancer cells, especially in coordination with CBP [15, 16]. However, the precise mechanisms of the CBP regulation of RFPL3-mediated hTERT activity and proliferation of breast cancer cells remain unknown, especially regarding the action of ASCs.

In this study, we found that ASCs promoted the proliferation of breast cancer cells. Mechanistically, we demonstrate that the activated MEK-ERK pathway mediated the nuclear recruitment of CBP in breast cancer cells cocultured with ASCs. Furthermore, CBP coordinated with RFPL-3 to subsequently activate hTERT transcription and telomerase activity. The inhibition of CBP and RFPL-3 suppressed the proliferation of breast cancer cells cocultured with ASCs following decreases in hTERT transcriptional activity in vitro and in vivo. Altogether, our study reveals a new regulatory mechanism and provides a new therapeutic target for treating breast cancer.

## 2. Method

### 2.1. Cell Lines, Stable Cell Lines, and Inhibitors

The MCF-10A, SKBR-3, MCF-7, and MDA-MB-231 cell lines were purchased from ATCC (Manassas, VA, USA). The medium were obtained from HyClone supplemented with 10% FBS (Gibco, USA). ASCs were prepared as our previous description [3]. Briefly, the pelleted cells being washed twice using PBS were labeled with rabbit polyclonal anti-c-kit antibody (Abcam, USA) and goat anti-rabbit IgG magnetic beads (Miltenyi Biotech Inc., USA). Anti-c-kit-labeled cells were further purified by fluorescence-activated cell sorting. The cells were incubated in 37°C humidifed incubator containing 5% CO_2_, and the medium was replaced for 2-3 days.

The scrambled nontarget control shRNA- or hTERT-expressing lentiviruses and the lentivirus particles for CBP short-hairpin RNA (shRNA) were purchased from GenePharma (Shanghai, China). The breast cancer cell lines were coinfected with CBP shRNA and the hTERT-expressing lentivirus to rescue hTERT expression.

Selumetinib and SP600125 (Selleckchem, USA) were diluted using DMSO to the final concentrations of 1 *μ*M and 10 *μ*M, respectively. SCF (R&D, USA) was used at a final concentration of 100 ng/ml.

### 2.2. Cell Viability Assay

Breast cancer cells were transfected with siRNA-CBP oligonucleotides or siRNA-RFPL3 oligonucleotides for 48 hours. In addition, Cells stably expressing CBP were treated with RFPL-3 siRNA or control siRNA, and the cells overexpressing RFPL-3 were cotransfected with CBP siRNA or control siRNA for 48 hours. Then, the cells (3 × 103 per well) were seeded into 96-well plates and cultured with the culture supernatant from ASCs for 24-96 hours. Thereafter, 10 *μ*l of CCK-8 solution per well was added, and the cells were further incubated for 3 h. The absorbance was measured using a microplate reader at 450 nm.

### 2.3. Cell Proliferation Assay

The cell proliferation was assessed by using dsDNA quantitation according to manufacturer's protocols as previously described [2]. After being cultured in the culture supernatant from ASCs for 24-96 hours, the transfected cells, including being transfected with siRNA-CBP oligonucleotides or siRNA-RFPL3 oligonucleotides, were washed with PBS and digested using Triton X-100. Then, the samples were centrifuged, the PicoGreen fluorescent reagent was added to the supernatant, and samples were transferred to 96-well plates. Fluorescence was detected with NanoDrop 3300 (Thermo Scientific, USA), and the dsDNA values were calculated according to known DNA standard curve.

### 2.4. Colony Formation Assays

The breast cancer cells (2 × 10^3^ per well) were cultured in 6-well plates with culture supernatant from ASCs. After 2 weeks, the colonies were fixed in MeOH and stained with crystal violet (Sigma, USA) for 30 min separately for counting.

### 2.5. Quantification of Cytokines/Chemokine

After being cultured for 1, 3, and 5 days, the cell culture supernatant was obtained and was simultaneously detected with MILLIPLEX MAP mouse cytokine/chemokine panel (Cat.MAGPMAG-24K, Millipore Corporation, USA) as previously described [17].

### 2.6. Immunofluorescence

Attached cells plated on 24-well plates were incubated with rat monoclonal to RFPL-3 (Abcam, USA) and donkey secondary antibody to rat (Alexa Fluor 488, Thermo Scientific, USA). Then, the cells were labeled with rabbit polyclonal to CBP (Abcam) and incubated with goat secondary antibody to rabbit (Alexa Fluor 647, Abcam). After being stained with DAPI for 1 min, the positive cells were observed using the confocal microscopy system (Yokogawa, Tokyo, Japan).

### 2.7. Transient Transfection

The vector including RFPL-3-expressing vectors, control vectors (GenePharma, Shanghai, China), and siRNA including RFPL-3 siRNA or nonspecific siRNA (GenePharma) were transfected into breast cancer cells using Lipofectamine 3000 (Invitrogen, USA) according to the manufacturer's protocol. In addition, breast cancer cells were transfected with pcDNA3.1-CBP or empty vector plasmids and CBP siRNA or nonspecific siRNA. After 48 hours, mRNA and protein were extracted, and telomerase activity was detected.

### 2.8. Dual-Luciferase Assay

Breast cancer cells overexpressing CBP and transfected with RFPL-3 siRNA or control siRNA were seeded into 96-well plates, and the luciferase plasmid including hTERT promoter was cotransfected into cells. In addition, the luciferase reporter plasmid was cotransfected into MDA-MB-231 cells stably expressing RFPL-3 and treated with control siRNA or CBP siRNA (Table S1). After twenty-four hours, cells were collected, and luciferase activity was analyzed as previously described [17].

### 2.9. RNA Extraction and qPCR

Total RNA (1 *μ*g) was extracted and reverse-transcripted into cDNA, and qPCR was performed as described before [3]. Amplification conditions were as follows: 95°C for 3 min (predenaturation), 35 cycles of denaturation at 95°C for 10 s, and extension at 60°C for 30 s (Table S2). The expression levels were analyzed with the 2^-△△Ct^ method.

### 2.10. Western Blotting

The protein samples were extracted and immunoblotted with primary antibodies against RFPL-3 (1 : 2000), hTERT (1 : 1000), CBP (1 : 1000), ERK1/2 (1 : 1000), p-ERK1/2 (pT202/pY204, 1 : 1000), c-JUN (1 : 2000), and p-cJUN (Ser63, 1 : 1000), overnight at 4°C, all obtained from Abcam. Then, the secondary antibodies were incubated at room temperature for 2 h, and the protein bands were visualized using an enhanced chemiluminescence detection kit (Beyotime Biotechnology, China) and western blot imaging system (Bio-Rad, USA).

### 2.11. Animal Studies

All the animal experiments were approved by the Animal Ethics Committee of Harbin Medical University. The BALB/c female nude mice aged four weeks were acquired from the Shanghai SLAC Laboratory Animal Co. All the care and use of experimental animals were in compliance with the National Institutes of Health's Guidelines. To establish the xenograft model, the 4-week-old BALB/c female nude mice (*n* = 5/group) were anesthetized with 1.2% Avertin (0.1 ml/10 g) and performed with subcutaneous orthotopic injection. The 10^6^ stable MDA-MB-231 cell lines, including those expressing (1) CBP-shRNA, (2) Scramble shRNA, (3) CBP shRNA + control empty vector, and (4) CBP-shRNA+hTERT, were resuspended in 200 *μ*l of PBS/Matrigel and injected into the mammary fourth fat pads of female nude mice with 10^6^ ASCs. The tumor growth was recorded twice/week, and the tumor volume was calculated as (*D*_max_ × *D*_min_^2^)/2. Twenty-one days after implantation, we sacrificed the mice and resected the tumors for further analysis.

### 2.12. ChIP Immunoprecipitation Assay

ChIP assays were performed as previously described [17]. In brief, the chromatin samples was sonicated from the cells after being fixed with 1% formaldehyde for 10 min and incubated with RFPL-3, CBP or IgG antibody. The 10% chromatin was used for the DNA input. The extracted DNA was assessed by PCR, and the primers as follows: hTERT, 5′-TGGCCCCTCCCTCGGGTTAC-3′, and 5′-CCAGGGCTTCCCACGTGCGC-3′. The amplified product was analyzed by agarose gel electrophoresis.

### 2.13. Streptavidin-Agarose Pull-down Assay

The streptavidin-agarose pull-down assay was performed to examine the transactivators binding to hTERT promoter DNA. A biotin-labeled hTERT promoter probe corresponding to the sequence -378 to +60 was synthesized by Sigma (St. Louis, USA) (sense: 5′-ACCCTGGGAGCGCGAGCGGC-3′; antisense: 5′-GGGGCGGGGTCCGCGCGGAG-3′). The mixture, including proteins from the nuclear extract (400 *μ*g), the DNA probe (4 *μ*g), and 40 *μ*l of streptavidin-agarose beads, was incubated with shaking for 2 h at room temperature to pull down the DNA-protein complex. Then, the bound proteins were collected for further analysis.

### 2.14. Telomerase Activity Assays

The telomerase PCR ELISA assay kit from Roche Applied Science (Shanghai, China) was used to analyze the telomerase activity according to the manufacturer's instructions.

### 2.15. Statistical Analysis

Each experiment was repeated in triplicate. The experimental data was analyzed, and visualized graphs were made using GraphPad Prism version 5.0 software. One-way ANOVA and Newman-Keuls post hoc tests were used to compare variances between groups. *P* value less than 0.05 was considered to be a significant difference.

## 3. Results

### 3.1. ASCs Enhance the Viability and Proliferation of Breast Cancer Cells following the Release of SCF

**Figure 1:**
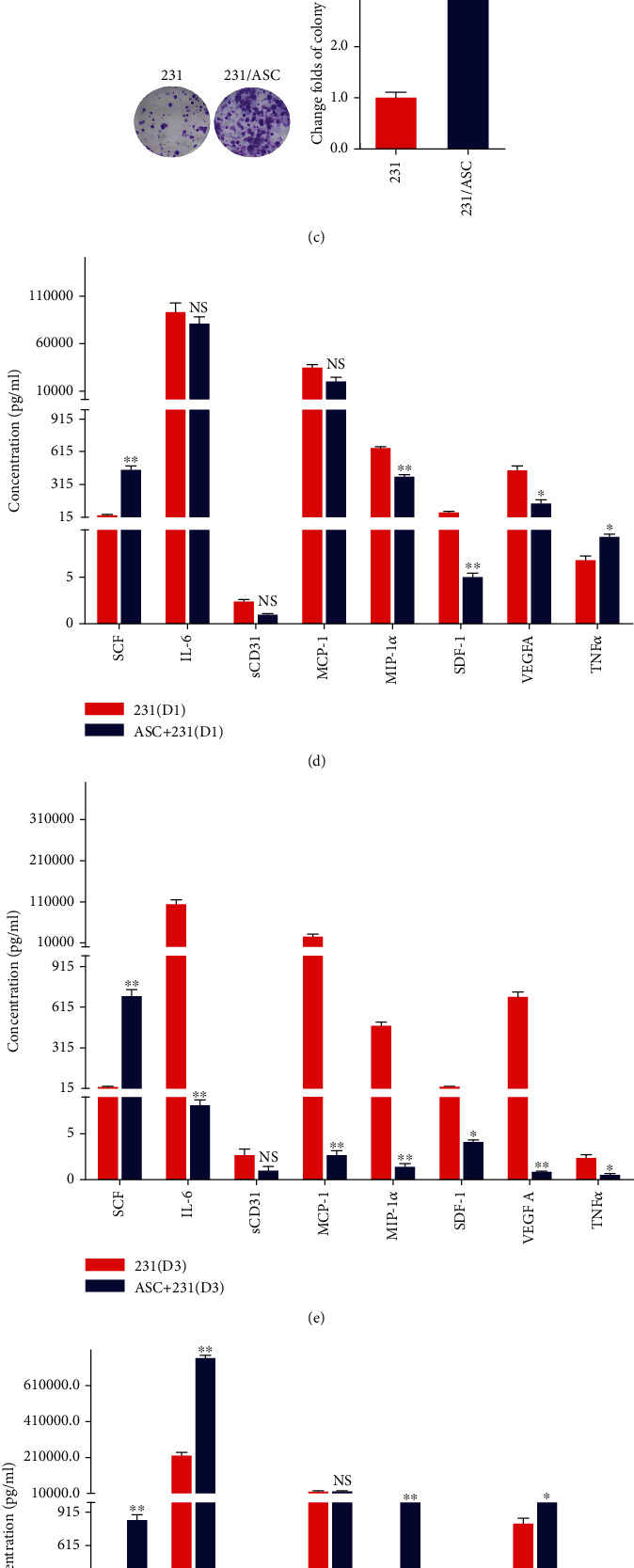
ASCs promote the viability, proliferation, and colony formation of breast cancer cells following the release of cytokines and chemokines. (a–c) Cell viability, proliferation, and colony formation were analyzed using CCK-8 assays, dsDNA quantification, and colony formation assays in MDA-MB-231 cells treated with culture supernatant from ASCs. (d–f) The release of cytokines and chemokines including SCF, IL-6, sCD31, MCP-1, MIP-1*α*, SDF-1, VEGFA, and TNF*α* in MDA-MB-231 cells cocultured with ASCs was analyzed using Milliplex MAP kit. ∗*P* < 0.05, ∗∗*P* < 0.01, ∗∗∗*P* < 0.001; NS: no significance.

To confirm the promotion of ASCs on the viability and proliferation of breast cancer cells, we treated the MDA-MB-231 breast cancer cells with the culture supernatant from ASCs. The results demonstrated that the viability, proliferation, and colony formation ability of MDA-MB-231 cells were significantly enhanced in the coculture with ASCs (Figures 1(a)–1(c)). Several cytokines and chemokines secreted from ASCs influence cancer cell behavior [17]. To reveal the molecular mechanisms by which ASCs promoted the proliferation of breast cancer cells, we analyzed the secretome in ASCs/MDA-MB-231 direct coculture, including SCF, sCD31, IL-6, monocyte chemotactic protein-1 (MCP-1), macrophage inflammatory protein-1*α* (MIP-1*α*), stromal cell-derived factor-1 (SDF-1), vascular endothelial growth factor A (VEGFA), and tumor necrosis factor *α* (TNF*α*). Quantification of secreted cytokines/chemokine has shown that an increase in the release of SCF from the coculture group comparing to the single culture groups (Figures 1(d)–1(f), Tables S3–5) and the secretion of SCF was also significantly greater in the ASC group than in the MDA-MB-231 group. However, the other factor levels were not significantly different among the groups (Figures 1(d)–1(f)). The same results were also presented in SKBR-3 breast cancer cells (Figure S1A–D).

### 3.2. MAPK-ERK Signaling Mediates the ASC-Stimulated Proliferation of Breast Cancer Cells by CBP Recruitment

**Figure 2:**
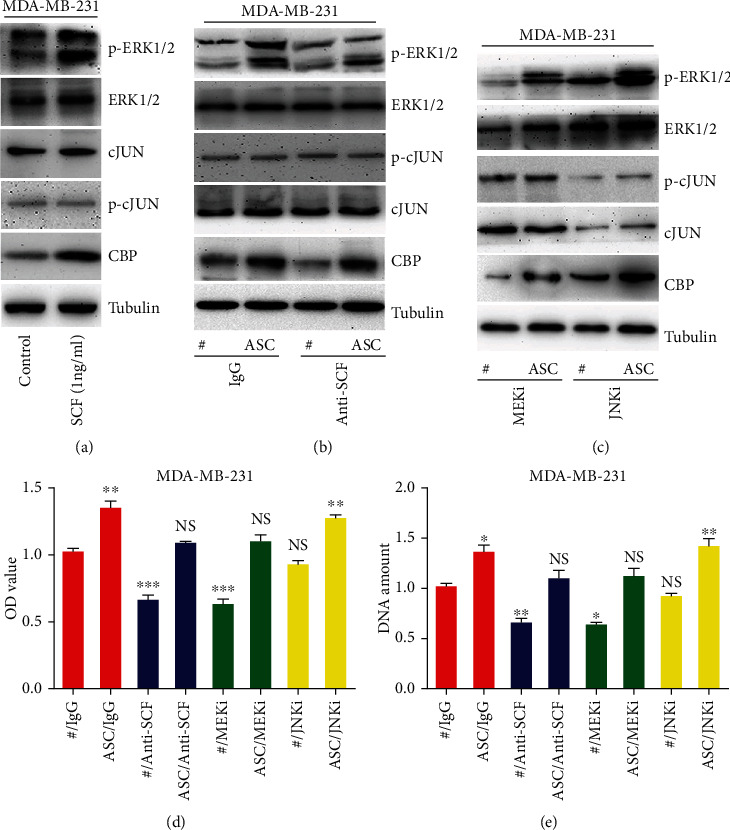
The effects of ASCs on MDA-MB-231 breast cancer cells are mediated by MEK-ERK-CBP signaling. (a) Western blot analysis of the association between MAPK pathways and CBP in MDA-MB-231 cells treated with ASC culture supernatant. (b–c) The indicated proteins in MDA-MB-231 cells induced by ASCs and the cell viability (d) and proliferation (e) were analyzed. The cells were pretreated with IgG- or anti-SCF-neutralizing antibody for 1 h, 1 *μ*M selumetinib (MEKi), and 10 *μ*M SP600125 (JNKi) for 24 hours. The results were normalized; the cells that were treated with IgG were used as a positive control; and the value was set to 1. ∗*P* < 0.05, ∗∗*P* < 0.01.

SCF, as the only known kit ligand, binds to the c-kit receptors and subsequently activates a series of downstream pathways including the MAPK pathways [18]. Therefore, we examined whether the pathways were activated in MDA-MB-231 cells treated with SCF and found that phosphorylated ERK (p-ERK) triggered CBP upregulation (Figure 2(a), Figure S2). Next, to further clarify the role of the MAPK-ERK pathway to CBP recruitment in the ASC-induced proliferation of breast cancer cells, we incubated the MDA-MB-231 cells with a MEK inhibitor, a JNK inhibitor, and an anti-SCF antibody and analyzed the changes in p-ERK and CBP expression levels using western blot. The results demonstrated that an anti-SCF antibody treatment significantly inhibited the CBP expression levels in the nuclei following the phospho-ERK1/2 decrease (Figures 2(b) and 2(c)). Moreover, treatment with MEK inhibitor decreased the ASC-stimulated CBP expression in MDA-MB-231 cells (Figure 2(c)). Interestingly, the JNK inhibitor did not significantly result in the inhibition of CBP expression in the nuclei (Figure 2(c)). Furthermore, the ASC-stimulated viability and proliferation of breast cancer cells were abrogated by the anti-SCF antibody and MEK inhibitor (Figures 2(d) and 2(e), Figure S3A–B). These results indicated that the SCF-MEK-ERK pathways activated CBP recruitment, which might mediate the ASC-stimulated proliferation of breast cancer cells.

### 3.3. CBP Coordination with RFPL-3 Activates the hTERT Transcription in Breast Cancer Cells

**Figure 3:**
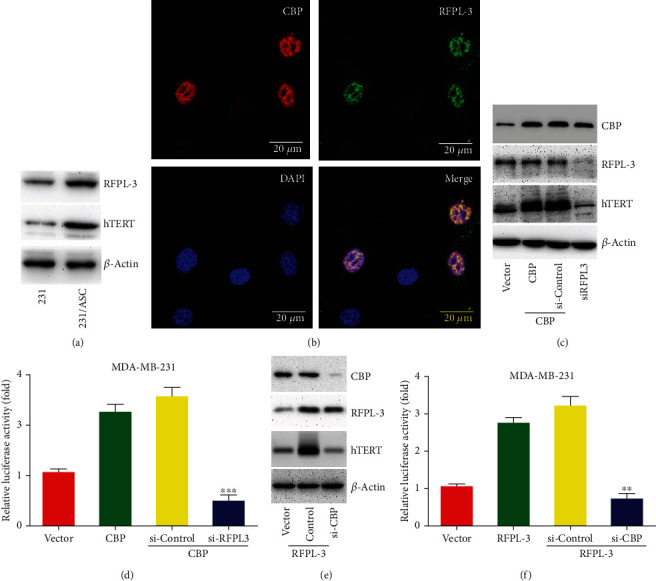
CBP coordinated with RFPL-3 to coregulate hTERT transcriptional activity in breast cancer cells. (a) MDA-MB-231 cells were treated with ASC culture supernatant, and then, the expression levels of RFPL-3 and hTERT protein were analyzed by western blot. (b) The localization of CBP and RFPL-3 in MDA-MB-231 cells was confirmed by confocal microscopy. (c) MDA-MB-231 cells were cotransfected with CBP vectors and nonspecific siRNA or RFPL-3 siRNA, and then, the expression levels of CBP, RFPL-3, and hTERT were detected using western blot. (d) MDA-MB-231 cells with CBP upregulation were co-transfected with RFPL-3 siRNA or control siRNA and hTERT-luciferase plasmids, and the relative luciferase activity was examined. (e) MDA-MB-231 cells were co-transfected with RFPL-3 vectors and CBP-specific siRNA or nonspecific siRNA, and then, the expression levels of RFPL-3, CBP, and hTERT were evaluated by western blot. (f) CBP-specific siRNA and hTERT promoter-driven luciferase plasmids were co-transfected into MDA-MB-231 cells overexpressing RFPL-3, and then, the relative luciferase activity was analyzed in the cells.∗∗*P* < 0.01, ∗∗∗*P* < 0.001.

It has been reported that CBP, as a transcriptional coactivator, could coordinate with RFPL-3 and regulate the hTERT transcriptional activation in lung cancer [16], and our results shown higher expression levels of RFPL-3 and hTERT in the indirect coculture of MDA-MB-231 cells with ASCs (Figure 3(a)) and shown that RFPL-3 was localized in the nuclei of MDA-MB-231 cells, with CBP (Figure 3(b)). To further investigate whether CBP coordinated with RFPL-3 to regulate hTERT transcription, cotransfection was performed with an hTERT promoter-driven luciferase plasmid and nonspecific siRNA, RFPL-3-specific siRNA or RFPL-3-specific siRNA in MDA-MB-231 cells, and SKBR-3 stably expressing CBP. We found that overexpression of CBP markedly increased hTERT upregulation, but RFPL-3 knockdown downregulated the elevated expression of hTERT without affecting CBP expression (Figure 3(c)). Similarly, the RFPL-3 knockdown caused a decline in hTERT promoter activity in MDA-MB-231 and SKBR-3 cells with CBP overexpression (Figure 3(d), Figure S4A). By contrast, the knockdown of CBP markedly decreased the hTERT upregulation caused by RFPL-3 overexpression but had no effect on the expression of RFPL-3 (Figure 3(e)). Furthermore, cells with CBP knockdown showed significantly attenuated hTERT promoter activity compared with cells overexpressing RFPL-3 (Figure 3(f), Figure S4B). These results suggested that CBP coordination with RFPL-3 activates the hTERT transcriptional activity in breast cancer cells.

### 3.4. Depletion of CBP and RFPL-3 Inhibits the ASC-Induced Breast Cancer Cells Proliferation by Downregulating hTERT Expression In Vitro and In Vivo

**Figure 4:**
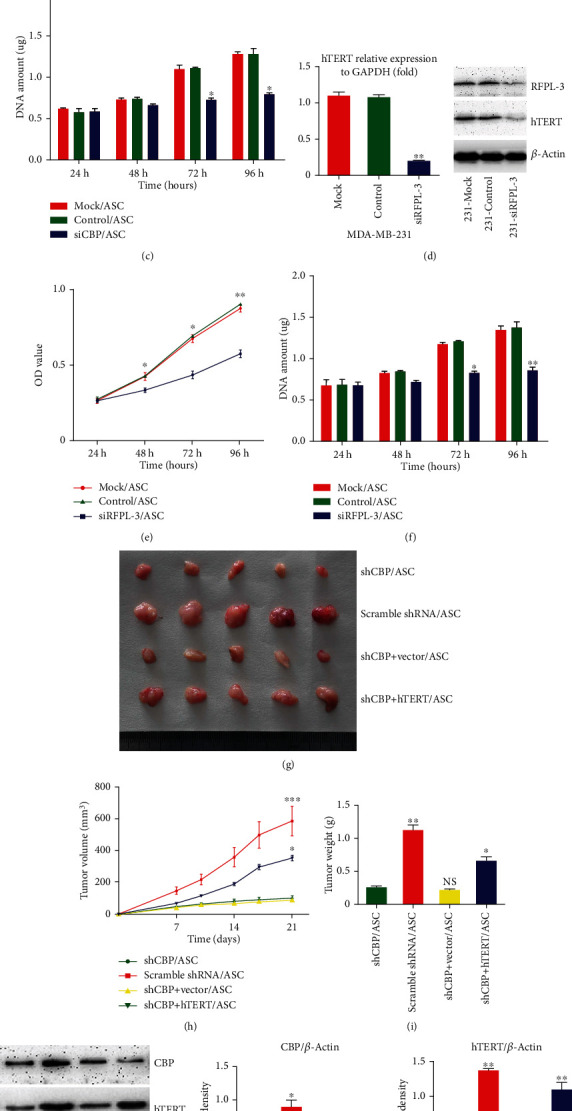
Inhibition of CBP and RFPL-3 suppresses the proliferation of breast cancer cells with cocultured ASCs following decreases in hTERT transcription in vitro and in vivo. (a) Real-time PCR and western blot analysis of the expression of hTERT mRNA and protein in MDA-MB-231 cells transfected with CBP siRNA and nonspecific siRNA. (b–c) The transfected MDA-MB-231 cells were treated with culture supernatant from ASCs, and the cell viability and proliferation were analyzed using CCK-8 assays and dsDNA quantification. (d) MDA-MB-231 cells were transfected with RFPL-3 siRNA and nonspecific siRNA, and then, hTERT mRNA and protein were examined using real-time-PCR and Western blot. (e–f) MDA-MB-231 cells transfected as above were treated with culture supernatant from ASCs, and then, the cell viability and proliferation were examined by CCK-8 assays and dsDNA quantification. (g) Tumor grafts up to 21 days after nude mice were injected with ASCs and cell lines stably expressing CBP shRNA, scramble shRNA, CBP shRNA + empty vector, and CBP shRNA + hTERT. (h) Tumor growth curves in nude mice. (i) The mean tumor weights 21 days after coinjection. (J) Western blot analysis of the expression of CBP and hTERT in tumor xenografts. ∗*P* < 0.05, ∗∗*P* < 0.01, ∗∗∗*P* < 0.001.

To further confirm whether CBP and RFPL-3 activated hTERT transcription, CBP-specific siRNA or RFPL-3-specific siRNA was used to knock down CBP or RFPL-3 expression, respectively, in SKBR-3 and MDA-MB-231 breast cancer cells. As shown in Figure 4(a) and Figure S5A, the downregulation of CBP results in a significant decrease in hTERT expression in MDA-MB-231 breast cancer cells. Besides, silencing CBP suppressed the ASC-induced proliferation in breast cancer cells (Figures 4(b) and 4(c), Figure S5B–C). Meanwhile, the inhibition of RFPL-3 led to the marked downregulation of hTERT expression and the promotion of proliferation in breast cancer cells cocultured with ASCs (Figures 4(d)–4(f), Figure S5D–F).

As a coactivator of hTERT, CBP was clearly involved in the ASC-induced proliferation of breast cancer cells in vitro; to further explore the mechanisms involved in the process of ASC-induced tumor growth, we constructed a series of stable cell lines expressing CBP shRNA, scramble shRNA, CBP shRNA + empty vector, or CBP shRNA + hTERT and performed subcutaneous coinjection of these cells and ASCs into nude mice. The results show that tumor growth was inhibited by the blockade of CBP in the mouse model (Figures 4(g)–4(i)). However, overexpression of hTERT in combination with CBP knockdown partially restored the tumor growth (Figures 4(g)–4(i)). The results were confirmed by western blots using xenograft tumor tissue (Figure 4(j)). Taken together, our findings clearly demonstrate that the depletion of CBP and RFPL-3 inhibits ASC-induced proliferation of breast cancer cells through the suppression of hTERT.

### 3.5. ASC Induction of CBP Upregulation Enhances Telomerase Activity by Activating the Binding of RFPL-3 to the hTERT Promoter Region in Breast Cancer Cells

**Figure 5:**
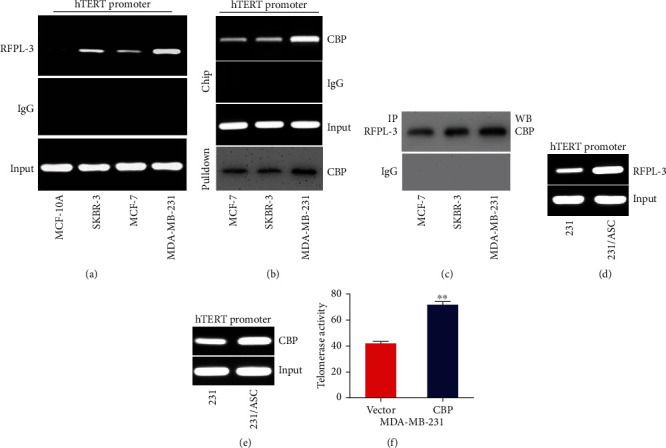
ASC induction of CBP upregulation enhances telomerase activity by activating the binding of RFPL-3 to the hTERT promoter region in breast cancer cells. (a) Chromatin immunoprecipitation assays were performed using anti-RFPL3 in normal breast cells and breast cancer cells. The amplified products of the hTERT promoter were analyzed by agarose gel electrophoresis. IgG was used as a negative control. (b) Chromatin immunoprecipitation analysis of the proteins bound to the hTERT promoter was performed using anti-CBP in breast cancer cells, and then, the pulled-down protein complex was examined by immunoblotting using anti-CBP. (c) The immunoprecipitation assay of nuclear extracts from breast cancer cells was carried out using anti-RFPL3, and then, the complexes were analyzed by immunoblotting using anti-CBP. (d–e) Chromatin immunoprecipitation analysis of the proteins bound to the hTERT promoter was performed using anti-RFPL3 and anti-CBP in breast cancer cells cocultured with ASCs. (f) The telomerase activity in MDA-MB-231 cells transfected with CBP vectors was measured using a TeloTAGGG telomerase PCR ELISAs. ∗∗*P* < 0.01.

A previous study showed that RFPL-3 could bind to the hTERT promoter and reactivate telomerase activity in lung cancer cells [15]. Accordingly, to detect the proteins on the hTERT promoter in breast cancer cells, we performed a ChIP assay and found that the RFPL-3 proteins bound to the hTERT promoter region in breast cancer cells, especially MDA-MB-231 cells, were higher than that in normal breast cells (Figure 5(a), Figure S6A). In addition, we also found by immunoprecipitation and pull-down analysis that CBP did indeed bind to the hTERT promoter and interact directly with RFPL-3 (Figures 5(b) and 5(c), Figure S6B). And, we found that the binding of RFPL-3 and CBP to the hTERT promoter was significantly increased in the MDA-MB-231 cells cocultured with ASCs compared to that in the untreated cells (Figures 5(d) and 5(e), Figure S6C–D). These results indicated that ASC induction of CBP upregulation mediated the binding of RFPL-3 to the hTERT promoter in breast cancer cells. To further examine whether the upregulation of CBP activated telomerase activity, the TeloTAGGG telomerase PCR ELISA was performed in MDA-MB-231 cells stably expressing CBP. The overexpression of CBP markedly upregulated the telomerase activity in MDA-MB-231 cells (Figure 5(f)). These results confirmed that ASC induction of CBP regulated the binding of RFPL-3 to the hTERT promoter region and subsequently activated telomerase activity in breast cancer cells.

## 4. Discussion

ASCs, with the abilities of differentiation and regeneration, have the potential to affect tumor growth after mastectomy [19], and we have reported that ASCs can favor tumourigenesis in breast cancer in our previous study [2]; however, it is not clear about the precise molecular mechanisms. The c-kit signaling network is involved in cell differentiation and proliferation [20], and the expression of SCF/c-kit is upregulated in the process of ASC-stimulated proliferation of breast cancer cell [2]. The activation of SCF/c-kit axis triggers cascade of MAPK pathways [21], of which the ERK pathway is generally responsible for cell differentiation and proliferation, whereas the JNK pathway is involved in apoptosis [22]. Additionally, the phospho-ERK translocation from the cytoplasm to the nucleus stimulates the activity of transcription factors such as STAT1/3, Pax6, and CREB [23–25]. In addition, CBP expression is markedly increased in breast cancer cells treated with cancer-associated adipose tissue [8]. Our results clearly confirmed that p-ERK is positively associated with SCF and CBP in ASC-induced breast cancer cell proliferation.

CBP can bind with transcription factors and activate transcription by acetylating histones and other regulatory proteins in tumourigenesis [26]. Meanwhile, RFPL-3 protein, which are most similar to the ret finger protein, contains RING, B30-2, and coiled-coil domains and interacts with other transcription factors [14]. Previous research has reported a synergistic effect of CBP and RFPL-3 in lung cancer [16]. Given this background, we explored whether CBP and RFPL-3 could coregulate hTERT transcription in the ASC-stimulated breast cancer cell proliferation. In line with our hypothesis, the results showed that overexpressed CBP did indeed regulate the ASC-stimulated breast cancer cell proliferation by activating the binding of RFPL-3 to the hTERT promoter region and enhancing telomerase activity.

Telomerase is involved in tumourigenesis-related signaling pathways in addition to telomere maintenance [27]. Telomerase reactivation or upregulation is governed by telomerase reverse transcriptase (TERT) expression [28, 29]. The hTERT promoter core contains GC boxes (GGGCGG) and E boxes (5′-CACGTG-3′), which provide binding sites for multiple transcription factors [30]. Some enhancer-binding proteins, such as zinc finger transcription factors, NF-*κ*B and c-Myc, bind to their respective sites and regulate hTERT transcription in different cellular contexts [31]. Herein, we only explore the role of CBP, not RFPL-3 in vivo because we find that the RFPL-3 protein cannot fully regulate hTERT expression, suggesting that other factors may regulate proliferation, such as the transcription factor activator protein-2 (AP-2), which is expressed in many human breast cancer cell lines and can activate c-erbB-2 and estrogen receptor promoters and increase their proliferation [32], or the others factors interfere with the function of RFPL-3, such as the Importin 13 (IPO13) can mediate the nuclear import of RFPL-3 through a functional NLS within RFPL-3 and subsequent hTERT expression upregulation [33].

**Figure 6:**
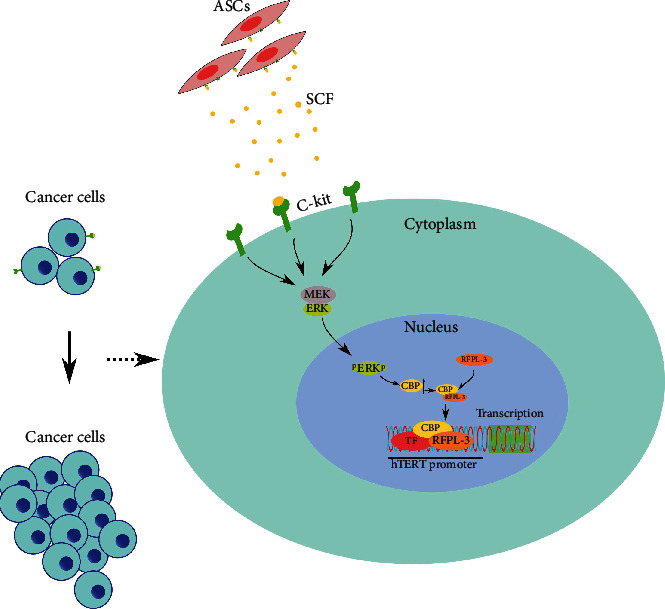
Overview of the proposed pathways for ASCs-induced proliferation of breast cancer cells.

In summary, our findings have shown that CBP coordinates with RFPL-3 to promote ASC-induced breast cancer cell proliferation by anchoring to the hTERT promoter and upregulating telomerase activity, which is activated by the MAPK/ERK signaling pathway (Figure 6). Hence, our studies uncover that the interference with MAPK/ERK/CBP-RFPL-3/hTERT pathway may be used as a potential therapeutic strategy to reduce ASC-stimulated proliferation of breast cancer cells.

## Data Availability

The data used to support the findings of this study are available from the corresponding author upon request.

## Supplementary Materials

Supplementary Materials The supplementary including the results in SKBR-3 breast cancer cells and the density analysis in western blot and CHIP assay. Figure S1: ASCs promote the viability, proliferation and colony formation of SKBR-3 breast cancer cells following the release of cytokines and chemokines. (A–C) Cell viability, proliferation, and colony formation were analyzed using CCK-8 assays, dsDNA quantification, and colony formation assays in SKBR-3 cells treated with culture supernatant from ASCs. (D) The release of cytokines and chemokines including SCF, IL-6, sCD31, MCP-1, MIP-1*α*, SDF-1, VEGFA, and TNF*α* in SKBR-3 cells cocultured five days with ASCs was analyzed using Milliplex MAP kit. ∗*P* < 0.05, ∗∗*P* < 0.01, ∗∗∗*P* < 0.001, NS: no significance. Figure S2: The expression of phosphorylated ERK and CBP in MDA-MB-231 cells treated with SCF (1 ng/ml). (A–B) Density assay shows the expression of p-ERK1/2 and CBP in MDA-MB-231 cells were activated by c-Kit+ ASCs using Image-Pro Plus 6.0 software. ∗*P* < 0.05. Figure S3: The SKBR-3 cells were pretreated with IgG or anti-SCF-neutralizing antibody for 1 h, 1 *μ*M selumetinib (MEKi), and 10 *μ*M SP600125 (JNKi) for 24 hours, and then, the cells' viability (A) and proliferation (B) were analyzed. The results were normalized, the cells that were treated with IgG were used as a positive control, and the value was set to 1. ∗*P* < 0.05, ∗∗*P* < 0.01. Figure S4: CBP coordinated with RFPL-3 to coregulate hTERT transcriptional activity in breast cancer cells. (A) The SKBR-3 cells with CBP upregulation were cotransfected with RFPL-3 siRNA or control siRNA and hTERT-luciferase plasmids, and the relative luciferase activity was examined. (B) CBP-specific siRNA and hTERT promoter-driven luciferase plasmids were cotransfected into SKBR-3 cells overexpressing RFPL-3, and then, the relative luciferase activity was analyzed in the cells. ∗∗*P* < 0.01, ∗∗∗*P* < 0.001. Figure S5: Inhibition of CBP and RFPL-3 suppresses the proliferation of breast cancer cells cocultured with ASCs following decreases in hTERT transcription. (A) Real-time PCR and western blot analysis of the expression of hTERT mRNA and protein in SKBR-3 cells transfected with CBP siRNA and nonspecific siRNA. (B–C) The transfected SKBR-3 cells were treated with culture supernatant from ASCs, and the cell viability and proliferation were analyzed using CCK-8 assays and dsDNA quantification. (D) SKBR-3 cells were transfected with RFPL-3 siRNA and nonspecific siRNA, and then, hTERT mRNA and protein were examined using real-time PCR and western blot. (E–F) SKBR-3 cells transfected as above were treated with culture supernatant from ASCs, and then, the cell viability and proliferation were examined by CCK-8 assays and dsDNA quantification. Figure S6: Density analysis of the ChIP data. (A–B) Density assay shows the binding of RFPL-3 and CBP proteins on the hTERT promoter region in MDA-MB-231 breast cancer cells and was higher than that in MCF-7 and SKBR-3 breast cancer cells in ChIP assay. (C–D) The relative densities of the binding of RFPL-3 and CBP to the hTERT promoter in Figures 5(d) and 5(e) were analyzed using Image-Pro Plus 6.0 software. ∗∗*P* < 0.01, ∗∗∗*P* < 0.001. Table S1 The sequence of RNA oligonucleotides or shRNA. Table S2 The sequence of primer. Table S3 The release of cytokines in MDA-MB-231 cells cocultured one day with c-Kit+ ASCs. Table S4 The release of cytokines in MDA-MB-231 cells cocultured three days with c-Kit+ ASCs. Table S5 The release of cytokines in MDA-MB-231 cells cocultured five days with c-Kit+ ASCs.
