# Supplementary material for: MAPK/ERK-CBP-RFPL-3 Mediates Adipose-Derived Stem Cell-Induced Tumor Growth in Breast Cancer Cells by Activating Telomerase Reverse Transcriptase Expression
Source: Stem Cells Int. 2022 Jun 7;2022:8540535. doi: 10.1155/2022/8540535 (PMC9197637; doi:10.1155/2022/8540535)
Supplement: Supplementary Materials — The supplementary including the results in SKBR-3 breast cancer cells and the density analysis in western blot and CHIP assay. Figure S1: ASCs promote the viability, proliferation and colony formation of SKBR-3 breast cancer cells following the release of cytokines and chemokines. (A–C) Cell viability, proliferation, and colony formation were analyzed using CCK-8 assays, dsDNA quantification, and colony formation assays in SKBR-3 cells treated with culture supernatant from ASCs. (D) The release of cytokines and chemokines including SCF, IL-6, sCD31, MCP-1, MIP-1α, SDF-1, VEGFA, and TNFα in SKBR-3 cells cocultured five days with ASCs was analyzed using Milliplex MAP kit. ∗P < 0.05, ∗∗P < 0.01, ∗∗∗P < 0.001, NS: no significance. Figure S2: The expression of phosphorylated ERK and CBP in MDA-MB-231 cells treated with SCF (1 ng/ml). (A–B) Density assay shows the expression of p-ERK1/2 and CBP in MDA-MB-231 cells were activated by c-Kit+ ASCs using Image-Pro Plus 6.0 software. ∗P < 0.05. Figure S3: The SKBR-3 cells were pretreated with IgG or anti-SCF-neutralizing antibody for 1 h, 1 μM selumetinib (MEKi), and 10 μM SP600125 (JNKi) for 24 hours, and then, the cells' viability (A) and proliferation (B) were analyzed. The results were normalized, the cells that were treated with IgG were used as a positive control, and the value was set to 1. ∗P < 0.05, ∗∗P < 0.01. Figure S4: CBP coordinated with RFPL-3 to coregulate hTERT transcriptional activity in breast cancer cells. (A) The SKBR-3 cells with CBP upregulation were cotransfected with RFPL-3 siRNA or control siRNA and hTERT-luciferase plasmids, and the relative luciferase activity was examined. (B) CBP-specific siRNA and hTERT promoter-driven luciferase plasmids were cotransfected into SKBR-3 cells overexpressing RFPL-3, and then, the relative luciferase activity was analyzed in the cells. ∗∗P < 0.01, ∗∗∗P < 0.001. Figure S5: Inhibition of CBP and RFPL-3 suppresses the proliferation of breast cancer cells cocultured with [file 8540535.f1.zip › TABLE S1 (1).pdf]

RNA oligo or shRNA	Sequences
siRNA-RFPL-3	F:5'-GUGGGAACAAGCACAGAAUTT-3'
	R:5'-AUAUCCACUUGGAACUUCCTT-3'
si-CBP	F: 5'-GAGGUCGCGUUUACAUAAATT-3'
	R: 5'-UUUAUGUAAACGCGACCUCTT-3'
si-Control	F: 5'-UUCUCCGAACGUGUCACGUTT-3'
	R: 5'-ACGUGACACGUUCGGAGAATT-3'
shRNA-CBP	F:5'-GGACAGCACACCTCTAAGAAGCT
	CTGACAGGGTGTTTCGTCCTTTC-3'
	R:5'-CCATCTCTTGAATGGAATGTGGAG
	GTTGGGAGGGAAAGAGTGATC -3'
shRNA-control	F:5'TGCTGAAATGTACTGCGCGTGGAGACGTTTT
	GGCCACTGACTGACGTCTCCACGCAGTACATTT-3'
	R:5'CCTGAAATGTACTGCGTGGAGACGTCAGTC
	AGTGGCCAAAACGTCTCCACGCGCAGTACATTTC-3'

Note: F stands for forward; R stands for reverse.
